# Preclinical optimization of a diode laser-based clamp-free partial nephrectomy in a large animal model

**DOI:** 10.1038/s41598-023-35891-1

**Published:** 2023-06-07

**Authors:** Weslley S. Andrade, Fenny H. F. Tang, Antonio C. H. Mariotti, Marilia W. Mancini, Ivison X. Duarte, Eric A. Singer, Robert E. Weiss, Renata Pasqualini, Wadih Arap, Marco A. Arap

**Affiliations:** 1 Hospital Sírio-Libanês, São Paulo, SP Brazil; 2 Rutgers Cancer Institute of New Jersey, Newark, NJ USA; 3 Division of Cancer Biology, Department of Radiation Oncology, Rutgers New Jersey Medical School, Newark, NJ USA; 4 Research and Education Center for Phototherapy in Health Sciences (NUPEN), São Carlos, SP Brazil; 5 Patologika Laboratory, Aracaju, SE Brazil; 6 Rutgers Cancer Institute of New Jersey, New Brunswick, NJ USA; 7 Section of Urologic Oncology, Division of Urology, Department of Surgery, Rutgers Robert Wood Johnson Medical School, New Brunswick, NJ USA; 8 Division of Urology, Department of Surgery, Rutgers New Jersey Medical School, Newark, NJ USA; 9 Division of Hematology/Oncology, Department of Medicine, Rutgers New Jersey Medical School, Newark, NJ USA; 10 Department of Urology, University of São Paulo School of Medicine, São Paulo, SP Brazil

**Keywords:** Kidney, Lasers, LEDs and light sources

## Abstract

Kidney cancer is a common urologic malignancy with either laparoscopic (LPN) or robotic partial nephrectomy as therapeutic options of choice for localized tumors. However, renal resection and suturing are challenging steps of the procedure that can lead to complications such as prolonged warm ischemia, bleeding, and urinary fistulas. LPN with a diode laser is an efficient technique due to its cutting and/or coagulation attributes. Surprisingly, key laser features such as wavelength and power remain undefined. Using a large porcine model, we evaluated the laser range of wavelength and power in a clamp-free LPN and compared it to the established gold-standard LPN technique (i.e., cold-cutting and suturing). By analyzing surgery duration, bleeding, presence of urine leak, tissue damage related to the resected renal fragment and the remaining organ, hemoglobin levels, and renal function, we show that an optimized experimental diode laser clamp-free LPN (wavelength, 980 nm; power, 15 W) had shorter surgery time with less bleeding, and better postoperative renal function recovery when compared to the well-established technique. Together, our data indicate that partial nephrectomy with a diode laser clamp-free LPN technique is an improved alternative to the gold-standard technique. Therefore, translational clinical trials towards human patient applications are readily feasible.

## Introduction

In 2022 there were an estimated 79,000 new cases of kidney cancer, with 13,920 deaths attributed to renal cell carcinoma (RCC) in the US alone^1^. Surgery is the primary treatment for most patients, with partial nephrectomy (PN), also known as nephron-sparing surgery (NSS), representing the standard option of treatment for cT1 renal mass^2–6^. When compared with radical nephrectomy (i.e., resection of the entire kidney), PN preserves renal function while maintaining similar oncologic outcomes with decreased overall mortality, and reduced frequency of cardiovascular events^7–12^. Although laparoscopic and robotic PN (LPN and RPN, respectively) present technical challenges and require comprehensive optimization and a steep learning curve, it is becoming an attractive alternative to open PN (OPN) with benefits to patients being a small incision area with preservation of nephrons, less blood loss, lower morbidity, faster postoperative recovery, aesthetic benefits, and comparable oncologic and functional outcomes^13–20^. Traditionally, PN has been performed with vascular occlusion, allowing the surgeon to minimize bleeding and improve visualization of the tumor and the remaining renal surface after tumor resection. However, the temporary interruption of arterial flow may lead to ischemic injury of the healthy renal parenchyma^21–26^. Therefore, there is an unmet need for novel techniques that allow NSS with reduced injuries to renal tissue due to clamp time ischemia, especially in patients with solitary kidneys or impaired renal function^7,27,28^.

**Figure 1:**
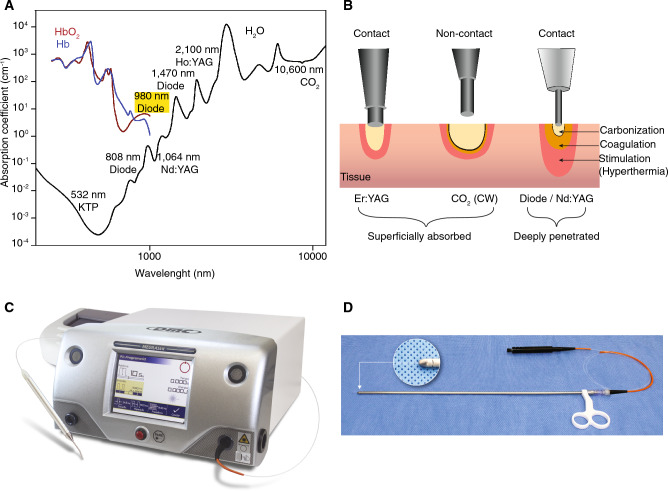
Laser source for PN. (**A**) Absorption spectra of water, hemoglobin (HbO_2_), and deoxyhemoglobin (Hb), the most abundant chromophores involved in the scope of laser partial nephrectomies. (**B**) Schematic representation of the lasers classification, according to penetration depth in biological tissue. CO_2_, carbon dioxide; CW, continuous wave; Er:YAG, erbium-doped yttrium–aluminium-garnet; Nd:YAG, neodymium-doped yttrium–aluminium-garnet. (**C**) Medilaser-DMC^®^ Diode Laser equipment. (**D**) Laparoscopic cannula—Model GN—DMC with 600 µm Fiber Optic (Introducer Kit for FO-DMC Catheter).

In surgical procedures, laser sources are used to emit a single, coherent, monochromatic light beam to cut, coagulate, or ablate tissue for a variety of clinical applications. Laser-assisted LPN uses the excellent coagulative properties of lasers to provide a bloodless tumor excision without the need for vascular clamping. The interaction between laser and tissue mainly depends on the type of laser (wavelength) and the components of the tissue interacting with the laser beam, such as water, chromophores, amino acids, and nucleic acids^29^. Different wavelengths have different absorptions in different types of tissue, and finding the optimal wavelength is crucial. PN using lasers employs the photothermal laser-tissue interaction, using continuous wave (CW) to pulsed lasers (milliseconds to a few seconds pulse widths), such as KTP (potassium-titanium-phosphate, 532 nm)^30^, diode lasers (980 nm and 1318 nm)^31–36^, holmium (Ho:YAG, 2100 nm)^37^, carbon dioxide (CO_2_)^38^, and thulium (Th:YAG and Diode Pumped Solid State Laser)^39,40^ lasers (Fig. 1A). The level of absorption is related to the depth of penetration and depends on the laser wavelength. According to the laser wavelength, they can be classified into two types: (i) superficially absorbed, such as CO_2_ and erbium (Er:YAG) lasers, in which the laser beam is absorbed in the superficial layer and does not penetrate or scatter deeply, and (ii) deeply penetrating lasers, such as neodymium-doped (Nd:YAG) and diode lasers, in which the laser beam penetrates and scatters deeply into the tissue (Fig. 1B).

In recent years, diode lasers were the focus of research regarding laser-assisted LPN due to their excellent cutting and coagulation features, and that they have been employed in humans and porcine models with a wide wavelength range within 442 and 2013 nm and laser power ranging from 15 to 150 Watts (W)^31,41–45^. Based on the absorption spectrum of the laser, clinicians determine which wavelength they should work with according to their purpose (Table 1). The ideal laser setup should provide precise and adequate tissue cutting and ablation without causing carbonization, splattering, or excessive smoke^42^. In our study, the diode laser was chosen over other light energies, as it is simultaneously absorbed by water and hemoglobin, which are the most characteristic and frequent chromophores in the renal tissue. Without the need for renal hilar clamping, surgeries were performed using a Medilaser DMC^®^ device (Fig. 1C) and a silica optic fiber inserted through a standard laparoscopic cannula (Fig. 1D). Here our primary goal was to conduct a prospective and comprehensive side-by-side study using a large animal model to evaluate the optimal wavelength and the optimal laser power of a diode laser light emitter in a clamp-free LPN technique that provided the most promising results in preclinical tests.

**Table 1:** Attributes of systems for use in laser-supported partial nephrectomy in patients.

Laser system	Laser type	Wavelength (nm)	Power on console (W)
RevoLix RevoLix 2 μm diode laser; LISA laser products, Katlenburg, Germany	Diode	2013	15–30^26^
Eraser, Rolle & Rolle, Salzburg, Austria	Diode	1318	1–100^27,31^
RevoLix^®^; LISA laser products, Katlenburg, Germany	Thulium	–	30–40^39^
Ceralas^®^ HPD, Biolitec AG, Jena, Germany	Diode	980	20–80^45^
Ceralas^®^ HPD DUAL, Biolitec AG, Jena, Germany	Diode	980 and 1470	20–80^45^

## Methods

### Experimental animals and study approval

This is an experimental study using a large porcine model (*Minipig BR*), as the pig is considered one of the most suitable animal models for renal procedures due to its structural anatomy and physiology that resembles the human kidney^46–49^. Typically provided by the vendor, thirteen healthy 7-month old female pigs with an average 12 kg weight were used. Following the principles of the Three Rs (Replacement, Reduction and Refinement), we randomly selected the first animal for a pilot study to determine the optimal parameters (wavelength and optical power) for the laser. The use of one animal provided a sample size of two kidneys, supplying the study with four kidney segments. The remaining twelve animals were randomly divided into four groups: one control group that underwent a standard partial nephrectomy technique using cold-cutting after mechanical renal clamping, and three laser groups with three different optical powers (10, 15 and 20 Watts, respectively). All animal experiments complied with the ARRIVE guidelines. All animal care and procedures were approved by the Institutional Animal Care and Utilization Committees of the Teaching and Research Institute of Sírio Libanês Hospital and performed in accordance with national legislation (Approval No. CEUA P 2016-01).

### Laser

In the experiment, we compared clampless laser laparoscopic partial nephrectomies to a standard cold-cutting procedure. A Medilaser Dual device yielding 980 nm + 1470 nm and Medilaser 450 nm—DMC^®^, Brazil, was used. A semiconductor laser beam 980 nm from a dual wavelength diode laser with 30 W continuous wave (CW) maximum output power, operating in both CW and pulsed mode, was used with optical powers of 10W, 15W and 20 W in CW operation and fiber contact mode. A silica optic fiber with a 600 μm nucleus—DMC was used and inserted through a 5 mm (mm) standard laparoscopic cannula introduced into the abdominal cavity by an ancillary trocar**.** During each resection procedure, the fiber was cleaned of the adhered tissue debris and cleaved as many times as needed, without significant impact on surgical time. The cleavage procedure consisted of prior fiber tip removal with scissors, followed by fiber polymer coating stripping and cleavage of the distal emitting point by a cleaving tool.

### Anesthetic induction and surgery technique

Animals were anesthetized with ketamine (5 mg/kg) and midazolam (0.5 mg/kg) intramuscularly and transferred from the vivarium to the preoperative table where venipuncture was performed for the administration of fluids and drugs. From the preoperative table, animals were moved to the operating room where anesthesia was induced by intravenous administration of 1% propofol (8 mg/kg). Then, they were orotracheally intubated and administered 2.5% isoflurane associated with 100% oxygen, with continuous infusion during the surgical procedure to maintain the anesthetic state. After anesthetic induction, the animal was placed in lateral decubitus, and shaving, asepsis, and antisepsis were performed. Carbon dioxide was used for abdominal insufflation during laparoscopy. During the laser LPN procedure, two surgical insufflators were used and, when necessary, one of the trocar valves was opened to allow removal of the smoke. Four laparoscopy trocars were introduced, located as follows: a 12 mm portal above the umbilical scar, where the camera was introduced; a 5 mm portal, lateral and superior to the camera portal, in the cranial direction (at the level of the hypochondrium); a 12 mm portal, laterally to the umbilical scar in the caudal direction, and another 12 mm portal, lateral and also superior to the camera portal, in the caudal direction, at the same distance from the 5 mm portal (Supplemental Fig. 3). These trocar positions provide the best triangulation of the instruments and prevent collision during dissection. In order to approach the kidney transperitoneally, the peritoneum on its lateral margin was incised and the kidney dissected with the assistance of hook forceps. A malleable ruler was introduced for the exact measurement of 2 cm of the LPN in each renal pole. During laser LPN, a 0.5–1 cm fiber-to-tissue distance was used, enhancing the visualization of the removed segment and allowing good target control.

For the pilot study, a compatible diode laser at moderate cutting speed (1–3 mm/s) in a continuous wave with settings of 450 nm (with 5 W and 10 W), 980 nm (10 W), 980 (15 W), as well as a dual wavelength combination 980 nm (15 W) + 1470 nm (3 W), simultaneously emitted through the same optical fiber, were tested. After defining the best minimal power (10 Watts) and wavelength (980 nm), we proceeded to the comparison of clampless laser partial nephrectomies to standard cold-cutting procedures. Animals were randomly divided into four groups, each with three animals: a control (standard technique) and three *laser* groups (10, 15 and 20 Watts, respectively). Laparoscopic partial nephrectomies were performed bilaterally, according to a previous described technique^50^. Briefly, pneumoperitoneum was achieved using a Veress needle and abdominal pressure set to 15-mmHg. Trocars were placed with visual control (one 10 mm for the camera and two 5 mm trocars for dissection) and kidneys were carefully dissected. A 2-cm fragment was dissected from each renal pole (superior and inferior), using a 15-mmHg abdominal pressure. At the end of the procedures, the pressure was set to 5 mmHg to evaluate potential bleeding. After hemostasis was confirmed, the renal segments were removed and weighed in a precise digital scale in order to ensure similarity among the samples (kidney segments).

In the control group, after dissection of the renal hilum, vessel loops were placed and laparoscopic forceps were inserted for the temporary occlusion of the renal artery and vein, taking care to avoid traction and iatrogenic vascular injuries. With the kidney under warm ischemia, LPN of the upper and lower pole of each kidney was performed (bilaterally) by applying a cold cut with scissors, measuring 2 cm, using the same ruler to certify the size of the renal incision. After complete resection of the renal pole, a traditional suture of the renal parenchyma with 3–0 polyglactin thread was performed for hemostasis, according to the standard technique performed in laparoscopic partial nephrectomies^51,52^. After renal suture, the pneumoperitoneum pressure (maintained at 15 mmHg during all procedures) was reduced to 5 mmHg for inspection of renal surface hemorrhages, which were cauterized with monopolar electrocautery without the need for any hemostatic agent. After each NPL, the vascular clamp was opened and the vessel loops were removed. The renal segments were removed by enlarging the incision in one of the access portals. The abdominal portals and skin were routinely sutured. No post-operative drains were placed, as animal suffering was to be reduced to a minimum, and a re-exploration was previously planned in all animals.

### Post-surgery maintenance

After surgery recovery, animals were returned to the vivarium and offered food and water. Immediately after surgery, animals received 50 mg of tramadol subcutaneously and 30 mg of flunixinmeglumine subcutaneously for two days. During the experimental procedures in the porcine model (n = 12 pigs), two animals died after the procedures. Both fatalities were deemed unrelated to the use of laser in surgery: one pig from the control group showed persistent bleeding during dissection of the renal vein and its caval insertion, which would ultimately allow arterial and polar dissection, especially in approaching the right kidney. The other pig from the 10 W group, died from an unrecognized lesion of the epigastric artery identified only after trocar removal, after the procedure; this epigastric lesion (presumed iatrogenic) was only recognized in the postmortem setting. With the exception of these two animals that died, all others were submitted to a new laparoscopy on the seventh postoperative day to assess the presence of adhesions and urinary fistulas through the intravenous injection of 5 mL of 1% methylene blue. Then, the animals were submitted to a bilateral nephrectomy to assess tissue damage in the remaining kidneys and then euthanized by intravenous injection of 25 mEq of potassium chloride.

### Pathological evaluation

Pathological evaluation of the renal fragments and kidneys was done by a certified pathologist (I.X.D.). Carbonization grade was assessed by measuring the tissue penetration (in mm) and the presence of coagulation necrosis. Surgical specimens were processed following conventional techniques for histological study, including the inclusion of the specimens in paraffin blocks, sectioning with a microtome at an average thickness of 5 μm, deparaffinization, and hematoxylin–eosin staining. Each renal segment was evaluated in 3 different areas, and at least 3 slides were made from each area. A total of 72 slides were evaluated per laser group and 60 slides for the controls, with 276 slides evaluated in total. A total of 276 slides were made (control group consisting of 60 slides and each laser power group of 10, 15 and 20 Watts consisting of 72 slides) for a complete evaluation of the removed segment and the remaining kidney. The patterns of necrosis and inflammatory response were evaluated, and the lesion areas were measured with a Nikon^®^ brand microscope, model E-200 eclipse, 40 × magnification lens.

### Statistical analysis

The sample size was estimated by reference to the average mean surgical time in a previous study^50^. The estimated mean surgical time was 8.71 min (SD 1.63 min) for the laser procedure group and 12.21 min (SD 2.98 min) for the standard surgery group, using an alpha error of 5% and a power of > 80%. Animal cohorts were estimated to be 8 observations (renal segments) for each group. Taking into consideration an estimated 20% loss, 12 observations were required per group (three animals per group), corresponding to a total of 12 animals needed to carry out the study.

For the exploratory data analysis, outliers were searched for variables related to the tissue damage caused by the laser and control group, and no extreme values were verified: the z-score variation for the measurements of microscopic lesions in the kidney segments and in the remaining kidneys ranged from − 2.15 to 1.64 and − 1.95 to 1.40 respectively. In the reliability analysis of the variables for the measurement of tissue damage caused by the laser and control group, a high reliability of the data was also verified, and Cronbach's alpha coefficient of the measurement of tissue damage was 0.994 for both the remaining kidney and the kidney segments. The four groups (10, 15, 20 W of power and control) were compared by multiple variance analysis (MANOVA), to verify which one presented the best settings in regard to the surgical time, being considered as the factor "the group" (powers 10, 15, 20 Watts and control) and dependent variables were the total and LPN surgical times, performed in each renal pole. For the variables bleeding volume, kidney segment weight and microscopic tissue damage, the variance analysis (ANOVA) with repeated measures was used as the first test, with the factor “the group” (power 10, 15, 20 Watts and control). The effect size was evaluated by the ɳ2. For intragroup comparison of laboratory dosages (hemoglobin, urea, creatinine) over time (preoperative, immediate postoperative, and on the seventh postoperative day of laparoscopic partial nephrectomy), Student's t-test for paired data was used. Statistical calculations were performed using GraphPad Prism 8. Statistical significance was set at *P* < 0.05.

## Results

### Optimization of wavelength and power of diode laser-assisted LPN in the porcine model

**Figure 2:**
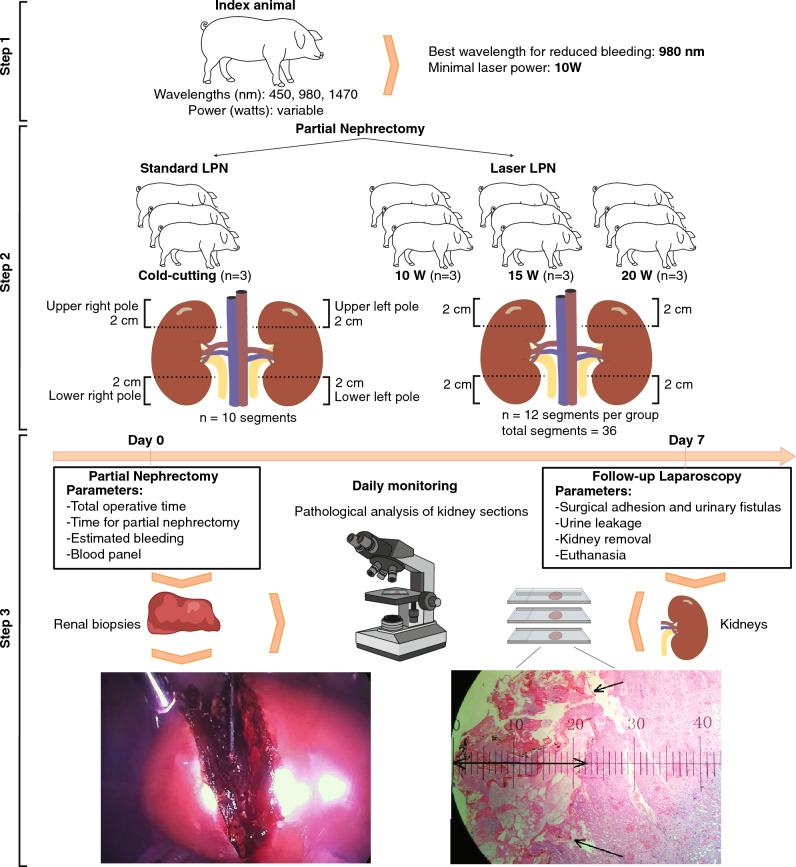
Schematic representation of diode LPN in pigs. Scheme of the approach used for the establishment of best settings (frequency and minimal optical power) in an index pig (step 1). LPN comparing standard technique with different laser powers (step 2), and work flow from preoperative to postoperative day (step 3).

Here we sought to standardize the best laser wavelength with a minimal power setting (W) for a diode laser to achieve cutting and coagulation proprieties that would mitigate the need for renal vascular clamping during laser-assisted LPN. First, we performed a pilot experiment where LPN was carried out to resect a 2 cm polar renal segment for both upper and lower poles from an index animal with an average kidney length of 11 cm, comparing the difference between: (i) 450 nm diode laser with settings of 5 W and 10 W, (ii) 980 nm diode laser with settings of 10 W and 15 W, and (iii) simultaneous combination of 980 nm (15 W) + 1470 nm (3 W), at moderate cutting speed (1–3 mm/s) and continuous wave (Fig. 2, step 1). When 450 nm (5W and 10W) and dual-wavelength combination 980 nm (15W) + 1470 nm (3W) were employed, we observed local bleeding during resection regardless of the power used, probably due to a shallower tissue depth penetration and faster ablation rate with insufficient interaction time for effective coagulation.

When the wavelength was changed to 980 nm during nephrectomy to avoid a possible hypovolemic shock in the animal, signs of coagulation were observed within the surgical timeframe, indicating that such a wavelength would be an ideal starting point for further validations. However, bleeding was persistent until the adjustment to 10 W power, resulting in a successful cut and coagulation of the renal parenchyma without the need for clamping the hilum. Collectively, we found that the use of the 980 nm diode laser with a starting optical power of 10 W is appropriated for partial nephrectomy in comparison to the hemoglobin-specific diode laser 450 nm and water-specific diode laser 1470 nm and can be applied in partial nephrectomy procedures preventing unnecessary bleeding and clamping. By standardizing the ideal laser wavelength and power to obtain the best cut and coagulation properties, we were also able to reduce smoke formation during the surgeries. In addition, two carbon dioxide insufflators were used, and when necessary, one of the trocar valves was opened to remove the smoke generated during the process. This procedure was sufficient to allow good visualization of the surgical field with a minimum amount of smoke.

### Comparison of clampless laser LPN to the standard cold-cutting procedure

After optimizing the wavelength (980 nm) and the minimal power (10 W) conditions for the use of the diode laser, we performed a comparative analysis of clampless LPN using the 980 nm diode laser to the standard cold-cutting LPN, which consisted of renal hilum clamping, removal of renal segments with a cold cut using scissors, followed by suture. Animals with an average kidney length of 11 cm^53,54^ were divided into four groups, each with three animals: a control group (standard technique) and three laser groups: 10, 15, and 20 W, respectively (Fig. 2, step 2). During the surgeries, of a total of 12 animals, two animals died after the procedure: one pig from the control group showed persistent bleeding during renal vein/caval dissection and the other pig from the 10 W group, died from a lesion of the epigastric artery identified only after removing the trocar post-procedure. Both fatalities were not related to the use of laser in surgery. To evaluate adherence and the presence of urinary fistulas, the animals were kept alive until the seventh postoperative day (Fig. 2, step 3).

**Figure 3:**
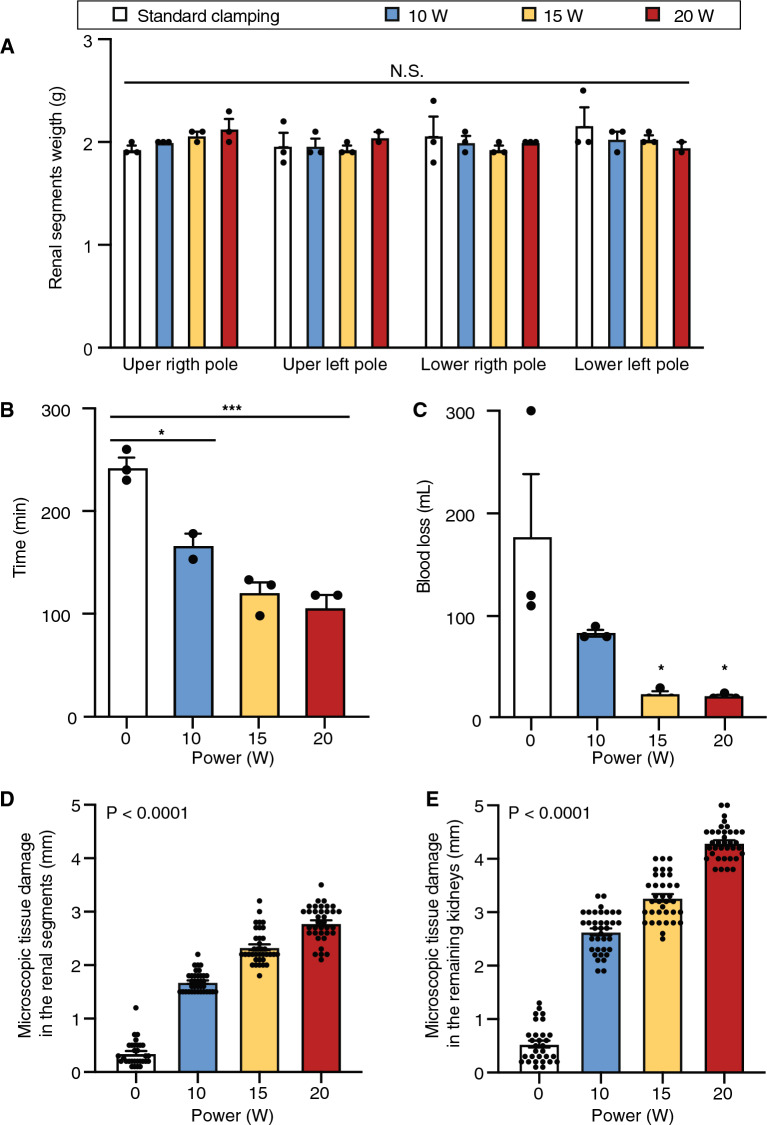
Surgical outcome and bleeding assessment. (**A**) Weight of the renal pole segments removed during laparoscopic partial nephrectomy. Data represent mean ± SEM. (**B**) Surgery total operative time (in minutes). Data represent mean ± SEM (*P < 0.05, ***P < 0.001) (**C**) Total intraoperative bleeding (in milliliters). Data represent mean ± SEM (*P < 0.05) (**D**) Microscopic tissue damage in the renal segments (in millimeters). Data represent mean ± SEM (****P < 0.0001). (**E**) Microscopic tissue damage in the remaining kidneys (in millimeters). Data represent mean  ± SEM (****P < 0.0001).

LPN were performed bilaterally, according to a previously described technique^50^. A 2-cm fragment (Supplemental Fig. 1) was removed from each renal pole and specimens were weighed on a precise digital scale, confirming that the size of the renal fragments was similar for all the groups, with no statistically significant difference between the groups (Fig. 3A). When the total operative time (in minutes)—defined as the period from the skin incision to the final skin suture—was analyzed, the control group presented a higher mean surgery time when compared to the laser LPN groups, and the higher the laser output power, the shorter the time (*P < 0.05, ***P < 0.001), with no significant difference observed between the 15 W and 20 W groups (Fig. 3B). The estimated bleeding was determined after each surgery by measuring the suctioned volume of blood in milliliters (mL): when intraoperative bleeding was analyzed, laser groups showed a significant reduction in the bleeding volume of the laser groups 15 W and 20 W in comparison to the control group (*P < 0.05), with no statistically significant difference observed between these two laser power groups (Fig. 3C). Together the analysis of total operative time and intraoperative bleeding indicated that the increase of the laser output power in the 980 nm diode laser shows a highly significant effect on surgery time and total bleeding volume, suggesting that 15 W is the ideal power that provided the best outcomes.

Next, we evaluated the presence of tissue damage in the renal segments and in the remaining kidneys by analyzing the patterns of necrosis and inflammatory response in the tissues. After pathological analysis of the renal fragments and remaining kidneys removed during surgery using laser and standard LPN, we observed that the depth of tissue damage increased proportionally with the increase in laser power. Tissue damage in the renal segments was significantly different between all the groups (****P < 0.0001), and directly proportional to the increase in laser power (Fig. 3D). When tissue damage in the remaining kidney was evaluated, we also observed significant differences between all the groups, with microscopic tissue damage in the remaining kidneys following the same pattern observed in the renal segments: increased tissue damage with increased power, with the 20 W power group presenting the highest tissue damage (****P < 0.0001) (Fig. 3E). On the seventh postoperative day, all animals in the 15 W and 20 W laser power groups showed adherence of ileum and/or colon to the kidney, in contrast to the animals in the control group, and only half of the animals in the 10 W laser power group showed adherence. Such adhesions did not prevent the dissection of the contralateral kidneys but admittedly added some technical challenges to the overall surgical procedure. Because the partial kidney resections were performed at varying distances of the renal calyces (ranging from a surgically safe distance to anatomically near or very near), on certain rare occasions, the urologists could not totally rule out the possibility that the corresponding calyces might have been inadvertently violated by the experimental procedure. However, despite the evident concern for iatrogenicity in the setting of a new experimental procedure, urinary fistulas were not observed in any of the experimental conditions, either by using methylene blue or by clinical observation during the postoperative recovery. Finally, no post-operative drains were placed, as animal suffering was to be reduced to a minimum.

### Impact of LPN on hematological parameters

**Figure 4:**
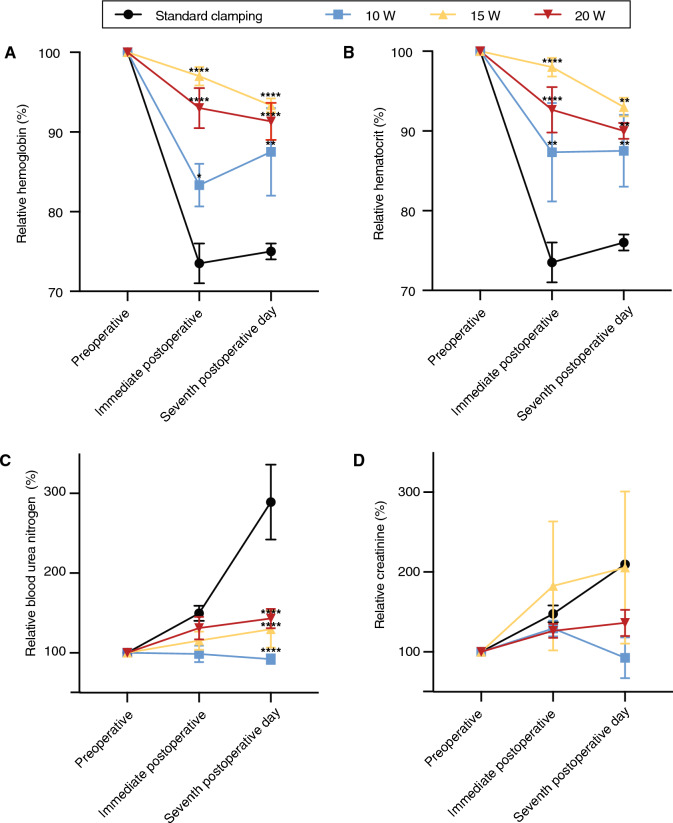
Hematological panel. Analysis of the levels of hemoglobin (**A**), hematocrit (**B**), blood urea nitrogen (**C**), and creatinine (**D**) in the preoperative, immediate postoperative and seventh postoperative day. Data represent mean ± SEM (*P < 0.05, **P < 0.01, ****P < 0.0001).

To assess the extent of blood loss and renal function after surgery, we also evaluated hemoglobin, hematocrit, creatinine, and blood urea nitrogen levels, comparing the variation of these parameters in the preoperative, immediate postoperative, and seventh postoperative day time points. Analysis of hemoglobin levels showed that animals treated with 15 W and 20 W laser power had less variation between preoperative, immediate postoperative, and seventh postoperative day, while in the control group, hemoglobin levels decreased to 73.5% immediately after surgery (*P < 0.05, **P < 0.01, ****P < 0.0001) (Fig. 4A). Analysis of the hematocrit levels followed a similar pattern observed in the hemoglobin levels, with less variation in the 15 W and 20 W laser power groups and a greater decrease in the control group (**P < 0.01, ****P < 0.0001) (Fig. 4B).

Next, we evaluated renal function based on the levels of blood urea nitrogen (BUN) and creatinine in the blood levels over time post-surgery. We observed that the control group showed high levels of blood urea immediately after the procedure and the difference significantly increased after seven days (****P < 0.0001), with no significant difference observed between the groups treated with the 980 nm diode laser regardless of the power that was used (Fig. 4C). Despite the postoperative difference in BUN measurement between laser groups and control group, no significant difference in creatinine levels was observed (Fig. 4D). Moreover, we also analyzed a panel of inflammatory or acute response cytokines and no difference was observed between the groups (Supplemental Fig. 2).

## Discussion

With the advances in diagnostic tests, such as computed tomography (CT) and magnetic resonance imaging (MRI), the incidence of patients diagnosed with RCC has rapidly increased over the past decades. In turn, there has been increased interest in understanding and preserving renal function in the setting of partial nephrectomy^55–60^. PN has emerged as an option for treatment of cT1 renal masses^61,62^ due to the higher risk of chronic kidney disease (CKD) development post radical nephrectomy^63,64^. Additionally, advances in methods to prevent ischemic renal damage is key to the final success of the PN procedure^65,66^.

In this study we developed a methodology for laser-assisted LPN that does not require vascular clamping or renal cooling, allowing simultaneous optimization of the cutting and coagulation processes. When compared to standard cold-cutting LPN, we demonstrate that the use of the 980 nm diode laser with 15 W power for LPN produced the best outcome by reducing the time of surgery, total intraoperative bleeding, tissue damage, and hematological alterations. Additionally, our data suggest that the use of the 980 nm diode laser may reduce or even eliminate the impact of warm ischemia and reduce the need for laparoscopic suture skills. The diode laser can also be used with the DaVinci^**®**^ robotic platform, as the optical fiber can be inserted via a standard 5 mm laparoscopic trocar, often used during robotic procedures. Together, our findings show the applicability of a diode laser light emitter for optimal cutting and coagulation features in LPN.

One of the main advantages of lasers in comparison with electrosurgery (mono and bipolar) is the control over energy distribution and the side thermal damage (STD)^67^. The main motivation for applying a coherent optical energy to partial nephrectomies is linked to the simultaneous excellent cutting and coagulation properties for small vessels in the renal parenchyma and avoiding the need for sutures, allowing a bloodless tumor excision without the need for renal artery clamping^68^. In addition to preserving the remaining healthy tissues from severe STD, lasers are also associated with lower inflammatory thermal effects and are therefore suitable for tissue sparing techniques.

The previously used neodymium-doped laser (Nd:YAG, 10,600 nm), which is poorly absorbed by water and hemoglobin chromophores, has been associated with profound optical depth and coagulative necrosis due to low absorption by water^69^. Thus, it has been largely replaced by the aforementioned laser systems due to their improved laser-tissue interactions arising from higher absorption by hemoglobin (namely, the KTP laser), hemoglobin and water (the diode lasers 980 nm and 1,318 nm) and high absorption by water (the holmium and thulium lasers). In a previous randomized study with a porcine model, the authors compared the KTP laser (532 nm) to conventional laparoscopy with LP; unfortunately, the lack of pathological analysis of the renal parenchyma limited the conclusions of that study^30^. Tissue damage is another central aspect to be considered when analyzing tumor specimens. A study by Drerup et al*.* in which a 1,318 nm diode laser and power ranging from 45 to 65 W was used in humans for partial nephrectomies, showed a 9% positive margin rate, with excessive carbonization of the specimens as the main limitation^31^. These concerns were addressed by our study: when 15 W optical power was used, the best results in terms of tissue penetration with efficient cut/coagulation capacity were obtained, while the 20 W group had an average of 0.5 mm deeper penetration. In fact, both laser power intensities had a good cutting/coagulation capacity and limited tissue damage (< 3 mm) and therefore could be utilized in human trials.

Surgical time was significantly reduced in all laser groups, and there were no conversions to open surgery in any procedure. This was probably due to the simplification of the procedure, in which no vascular clamping or renal suturing was necessary. Also, bleeding was minor in the 15 W and 20 W groups when compared to the control standard laparoscopic procedure. Additionally, we did not use any type of biological or synthetic glue or hemostatic agent as there were no significant acute or late bleedings. Ogan et al. using an 810 nm diode laser and 20 W power, eventually required the use of a biological glue^70^. In the settings used in this study, the 980 nm diode laser showed sufficient coagulation power to allow the resection of any renal segments in a human kidney, since intrarenal vessels usually have a diameter of 5 mm or less^71,72^.

When the renal function was analyzed taking into consideration BUN levels, all animals in the laser groups benefited from the lack of warm ischemia: on the seventh postoperative day, levels of BUN increased approximately 300% in the control group. This is in accordance with the study by Muramaki et al., in which 39.4% of patients submitted to conventional laparoscopic partial nephrectomies, with pedicle clamping, had postoperative renal insufficiency^73^. Other studies indicate that every warm ischemia minute adds additional risk for renal dysfunction in the postoperative period^74^, and warm ischemia is the most important single modifiable factor that regulates renal function in such patients^45,75^.

There are several studies discussing the complexity of the LPN procedure, as well as the surgical abilities required to achieve good results^76–79^. Unfortunately, two animals died from uncontrolled bleeding unrelated to the renal resection or renal hemostasis. The first animal died during caval dissection in order to access and control the renal vein, while the other had an epigastric artery lesion, identified only after removal of the trocar, which silently bled the animal to shock. This highlights the difficulties of renal LPN. It is important to emphasize that all procedures were done by two experienced laparoscopic surgeons and therefore we trust that the learning curve was not an issue and did not impact the standard partial nephrectomies performed in the control group.

A few limitations were observed in our study. First, the volume of tissue resected in our work (2 cm polar renal segment for both upper and lower poles) might not be appropriate for the analysis of urine leaks and collecting system injury. At the same time, studies that have removed larger portions have also encountered difficulties analyzing these parameters after the laser procedure. For example, Anderson et al. resected a large amount of tissue (30 g) and detected urine extravasation due to lasering into the collecting system^80^, and in the study performed by Eret et al. the authors were able to suture the collecting system, but they observed that the thermal injury prevented the collecting system from healing and detected delayed urine leaks^81^. Second, although we were able to detect the postoperative difference in BUN measurement between laser groups and control group, no significant difference was observed in the levels of creatinine, a common biomarker used to predict renal function. Serum creatinine concentration can be affected by factors other than glomerular filtration rate, such as age, gender, ethnicity, dietary habit, and muscle mass^82,83^, parameters that could have affected our analysis. There are many reports showing that serum creatinine and GFR estimation (eGFR) vary greatly in the acute to subacute phases post-surgery and only reach their final values several weeks after the procedure, however we were not allowed to keep the pigs alive for longer periods of time by the IACUC. Moreover, despite pigs being an established animal model for studying renal disease, surprisingly, there is no simple and reliable methodology to determine eGFR in swine^84^. Additionally, the translational value of these results might be challenging to incorporate in the absence of comparable eGFR after partial nephrectomies in human patients, which is not often used other than in cases of marginal renal function. Third, analysis of a panel of inflammatory or acute response cytokines in the immediate postoperative and seventh postoperative days showed no difference between the groups. Studies have recommended that systemic levels of the pro-inflammatory cytokines should be observed at 24–72 h (h) after the surgery. For example, it was found that during the first 24 h after resection there was a significant increase in the serum concentration of IL-6, which declined within the next 48–72 h, while the serum concentration of TNFα was highest at 18–24 h after the surgical procedure, and serum levels of IL-8 were markedly decreased from 12 h until 48 h postoperatively^85,86^. Fourth, renal tumors are all different, and some are easier than others to remove. We had to standardize partial nephrectomies in order to compare the use of laser to the standard technique. It is possible that for endophytic lesions, in which calyces are entered, the use of sutures may still be needed. Finally, the use of a diode laser 980 nm during the procedures generated a significant amount of smoke. This does not happen with the standard technique, but we were able to overcome it using a second insufflator. In humans, this effect may also be less evident as the human peritoneal cavity is larger than the pigs. Most renal tumors are treated using the robotic platform instead of a pure laparoscopic approach. We believe that, despite not using the robot, results would probably be similar, as both surgeons had extensive laparoscopic training. Additionally, the use of a diode laser is suitable for robotic PN, as the laser fiber can be inserted through any 5 mm trocar and therefore can be maneuvered with and without the robotic arms in the event of the surgery. Furthermore, if no suture is required, PN could be facilitated and probably available to a higher number of urologists and centers.

In conclusion, by employing a 980 nm diode laser with a 15 W power setup, we presented a safe and valid therapeutic method for laser-assisted LPN to remove small renal masses. The minimally invasive procedure provides various benefits, such as effective hemostasis, controlled bleeding, unaffected renal function, and prevention of complications. Further studies with larger sample size, additional time-points for blood sample collections, and longer follow-ups are required to confirm the findings of this preliminary study.

## Supplementary Information

Supplementary Figures.

## Data Availability

The data that support the findings of this study are available from the corresponding author upon reasonable request.
