# Supplementary material for: Preclinical optimization of a diode laser-based clamp-free partial nephrectomy in a large animal model
Source: Sci Rep. 2023 Jun 7;13:9237. doi: 10.1038/s41598-023-35891-1 (PMC10247752; doi:10.1038/s41598-023-35891-1)
Supplement: Supplementary file 1 — Supplementary Figures. [file 41598_2023_35891_MOESM1_ESM.pdf]

Preclinical optimization of a diode laser-based clamp-free partial nephrectomy in a large animal model

Weslley S. Andrade[1#], Fenny H. F. Tang[2,3#], Antonio C. H. Mariotti[1], Marilia W. Mancini[4], Ivison X. Duarte[5], Eric A. Singer[6,7], Robert E. Weiss[2,8], Renata Pasqualini[2,3], Wadih Arap[2,9*], and Marco A. Arap[1,10*]

[1]Hospital Sírio-Libanês, São Paulo, São Paulo, Brazil.

[2]Rutgers Cancer Institute of New Jersey, Newark, New Jersey, USA.

[3]Division of Cancer Biology, Department of Radiation Oncology, Rutgers New Jersey Medical School, Newark, New Jersey, USA.

[4]Research and Education Center for Phototherapy in Health Sciences (NUPEN), São Carlos, São Paulo, Brazil.

[5]Patologika Laboratory, Aracaju, Sergipe, Brazil.

[6]Rutgers Cancer Institute of New Jersey, New Brunswick, New Jersey, USA.

[7]Section of Urologic Oncology, Division of Urology, Department of Surgery, Rutgers Robert Wood Johnson Medical School, New Brunswick, New Jersey, USA.

[8]Division of Urology, Department of Surgery, Rutgers New Jersey Medical School, Newark, New Jersey, USA.

[9]Division of Hematology/Oncology, Department of Medicine, Rutgers New Jersey Medical School, Newark, New Jersey, USA.

[10]Department of Urology, University of São Paulo School of Medicine, São Paulo, São Paulo, Brazil

[#]Weslley S. Andrade and Fenny H. F. Tang contributed equally to this work.

***Address correspondence to:** Dr. Wadih Arap, Rutgers Cancer Institute of New Jersey, 205 South Orange Avenue, Suite B1101, Newark, New Jersey 07101, USA. Phone: 973-972-0366; Email: wadih.arap@rutgers.edu. Or to: Dr. Marco A. Arap, Hospital Sírio-Libanês, R. Dona Adma Jafet, 50, São Paulo, São Paulo 01308-050, Brazil. Phone: +55-11-3255-2933. Email: marcoarap@hotmail.com.

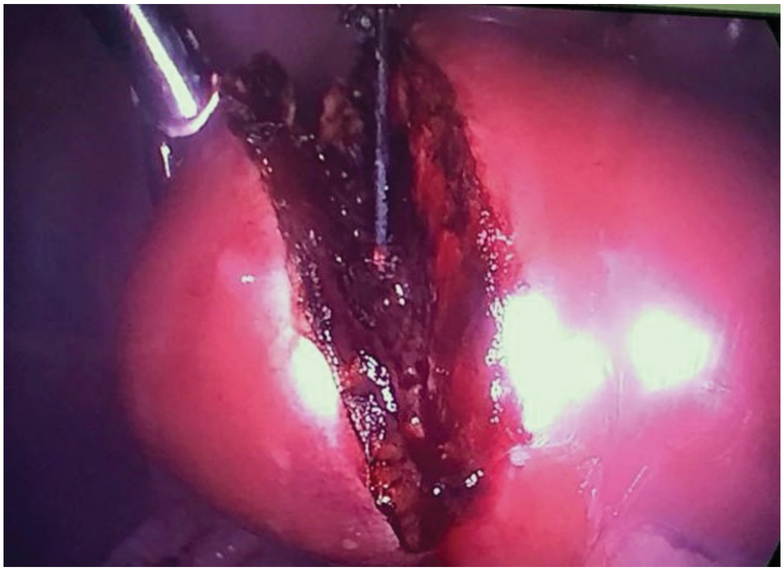

Supplemental Figure 1. Intraoperative aspect of the kidney. A 2 cm renal fragment was excised through laparoscopic partial nephrectomy with the use of the Diode laser at a power of 15 Watts.

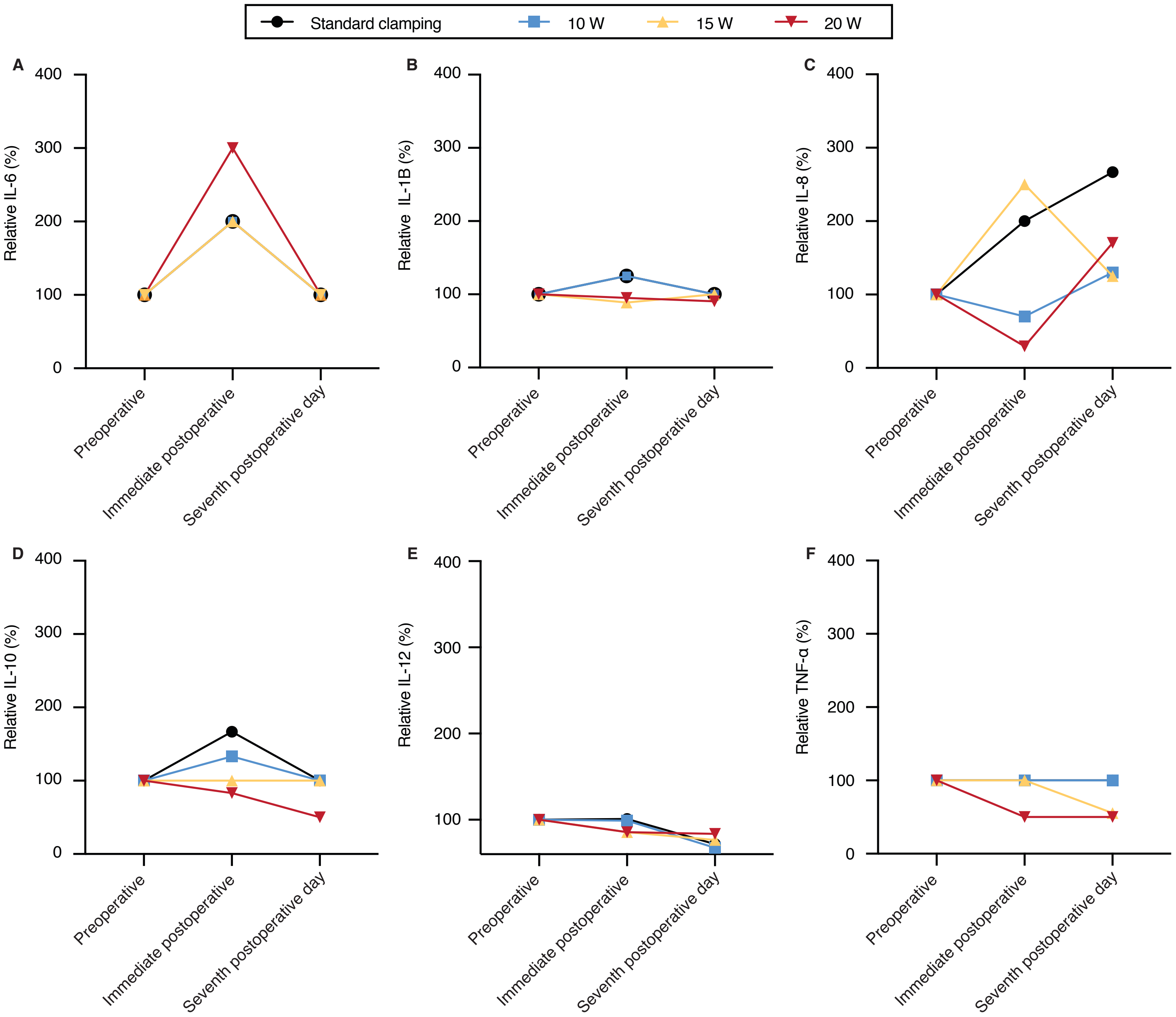

Supplemental Figure 2. Panel of inflammatory and acute response cytokines . Analysis of the levels of IL-6 (A), IL-1B (B), IL-8 (C), IL-10 (D), IL-12 (E), and TNF-α (F) in the preoperative, immediate postoperative and seventh postoperative day.

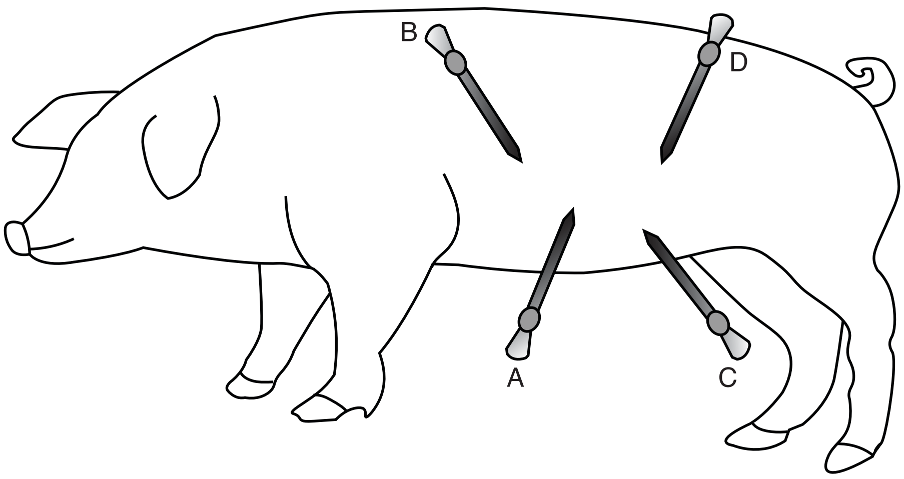

Supplemental Figure 3. Positioning of the laparoscopy trocars. A= 12 mm portal, placed above the umbilical scar (camera); B= 5 mm portal, lateral and superior to the camera portal, in the cranial direction (at the level of the hypochondrium); C= 12 mm portal, placed laterally to the umbilicus in the caudal direction; D= 12 mm portal, placed lateral and superior to the camera portal, in the caudal direction.
